# Supplementary material for: Content-rich biological network constructed by mining PubMed abstracts
Source: BMC Bioinformatics. 2004 Oct 8;5:147. doi: 10.1186/1471-2105-5-147 (PMC528731; doi:10.1186/1471-2105-5-147)
Supplement: Additional File 2 — The original results of the above study (non-essential files are deleted to keep the file size under the limit set by BMC bioinformatics). [file 1471-2105-5-147-S2.bz2 › chilibotAdditionalFile2/dip20/344ID10903799E4304/html/IL2RG_IL15.html]

 


 **IL2RG** and **IL15** 
  
Found 101 abstracts in PubMed, retrieved 20.  
 

 What does Google say? 
 PDF only 
| .edu only 

---

**Interactive relationship** (e.g. stimulation, inhibition, etc)

**Neutral relationship**- The receptors for interleukin 2 IL 2 and interleukin 15  [ **IL15** ]  IL 15  [ **IL15** ]  in T cells share the IL 2R beta subunit CD122 and gamma C  [ **IL2RG** ]  subunit but have private alpha subunits.  Ref: 12121224 Immunol Cell Biol, 2002
- The receptor consists of two chains IL 7 specific alpha chain CD127 and the common gamma c  [ **IL2RG** ]  chain CD132  [ **IL2RG** ]  which is a component of several other cytokine receptors IL 2, IL 4, IL 9 and IL 15  [ **IL15** ] .  Ref: 12644318 Immunol Lett, 2003
- The common gamma chain gamma c  [ **IL2RG** ]  is an indispensable subunit of the functional receptor complexes for IL 4, IL 7, IL 9, and IL 15  [ **IL15** ]  as well as IL 2.  Ref: 11418623 J Immunol, 2001
- Interleukin IL 2, IL 4, IL 7, IL 9, IL 15  [ **IL15** ] , and IL 21 form a family of cytokines based on their sharing the common cytokine receptor gamma chain, gamma c  [ **IL2RG** ] , which is mutated in X linked severe combined immunodeficiency SCID.  Ref: 12435740 J Biol Chem, 2003
- The genetic disorder results from mutations in the common gamma c  [ **IL2RG** ]  chain that participates in several cytokine receptors including the interleukin 2 Il 2, Il 4, Il 7, Il 9, Il 15  [ **IL15** ]  receptors.  Ref: 11265225 Pathol Biol Paris , 2001
- The common gamma chain gamma c  [ **IL2RG** ]  that functions both in ligand binding and signal transduction is a shared subunit of the multichain receptors for interleukin IL 2, IL 4, IL 7, IL 9, IL 15  [ **IL15** ] , and IL 21.  Ref: 11815609 J Biol Chem, 2002
- Our laboratory has identified an X linked severe combined immunodeficiency XSCID  [ **IL2RG** ]  in dogs that is the result of mutations in the common gamma chain gammac subunit of the interleukin 2 IL 2, IL 4, IL 7, IL 9, IL 15  [ **IL15** ] , and IL 21 receptors.  Ref: 12857971 Immunol Res, 2003
- Indeed, in BM precursors, neutralizing anti IL 15  [ **IL15** ]  monoclonal antibody mAb inhibits the expression of the gamma c  [ **IL2RG** ]  chain and of the chemokine stromal derived factor 1 SDF 1 but had no effect on vascular cell adhesion molecule 1 VCAM 1 and beta 1 integrin adhesion molecule expression.  Ref: 12623850 Blood< MedlineTA>Blood, 2003
- Dendritic cells DCs and DC derived cytokines allowed naive T cells to proliferate selectively in response to IL 4, and potently boosted the response of T CM to IL 7 and IL 15  [ **IL15** ]  by increasing the expression of the IL 2 IL 15Rbeta and the common gamma chain gamma c  [ **IL2RG** ] .  Ref: 11748273 J Exp Med, 2001

**Non-interactive relationship** (e.g. studied together, co-existance, homology, etc.)

- The chicken B cell marker ChB6, caspase 1, inhibitor of apoptosis protein 1 IAP 1, interleukin 15  [ **IL15** ]  receptor alpha chain IL 15Ralpha, interleukin 2 receptor gamma chain  [ **IL2RG** ]  IL 2Rgamma, and immunoglobulin supfamily gene ZOV3, as physiological candidate genes for chicken immune response, were selected to investigate associations with antibody kinetics to SRBC and killed B. abortus.  Ref: 12872968 Poult Sci, 2003
- The common cytokine receptor gamma chain gamma c  [ **IL2RG** ] , an essential component of the receptors for IL 2, IL 4, IL 7, IL 9, and IL 15  [ **IL15** ] , is critical for the development and function of lymphocytes.  Ref: 12093291 Biochemistry, 2002
- The cytokine interleukin 21 IL 21 is closely related to IL 2 and IL 15  [ **IL15** ] , and their receptors all share the common cytokine receptor gamma chain, gammac, which is mutated in humans with X linked severe combined immunodeficiency disease XSCID  [ **IL2RG** ] .  Ref: 12446913 Science, 2002
- In the present study, epitope mapping of antagonistic anti gamma c  [ **IL2RG** ]  monoclonal antibodies led to the identification of Asn 128 of mouse gamma c  [ **IL2RG** ]  that represents another potential contact residue that is required for binding IL 2, IL 7, and IL 15  [ **IL15** ]  but not IL 4.  Ref: 11815609 J Biol Chem, 2002
- These findings may be related to IL 15R alpha s ability to present IL 15  [ **IL15** ]  in trans to low affinity IL 15R beta gamma c  [ **IL2RG** ]  receptors on memory CD8 T cells.  Ref: 12671073 Proc Natl Acad Sci U S A, 2003
- Additionally, the fa fa rats expressed 84% lower levels of the gamma c  [ **IL2RG** ]  signaling receptor subunit than lean Zucker rats, suggesting this decrease may play a role in the lack of adipose tissue response to IL 15  [ **IL15** ]  in the fa fa genotype and lending further support for a direct action of IL 15  [ **IL15** ]  on adipose tissue.  Ref: 11960686 Biochim Biophys Acta, 2002
- In addition, ribonuclease protection assays RPAs were used to demonstrate that all three subunits gamma c  [ **IL2RG** ] , beta and alpha of the IL 15  [ **IL15** ]  receptor complex are expressed by rat and mouse WAT, suggesting that the effects of IL 15  [ **IL15** ]  on adipose tissue metabolism could be direct.  Ref: 11960686 Biochim Biophys Acta, 2002
- IL 21R shares the common gamma chain gamma c  [ **IL2RG** ]  with the receptors for IL 2, IL 4, IL 7, IL 9, and IL 15  [ **IL15** ] .  Ref: 12244150 J Immunol, 2002
- Conversely, in CB progenitors, anti IL 15  [ **IL15** ]  mAb inhibited VCAM 1 and beta 1 integrin expression without affecting gamma c  [ **IL2RG** ]  chain expression and, most important, up regulated SDF 1 expression.  Ref: 12623850 Blood< MedlineTA>Blood, 2002
- We studied the effect of cytokines on reproductive functions such as ovulation and pregnancy, with mutant mice lacking interleukin 2 receptor gamma  [ **IL2RG** ]  IL 2Rgamma, the so called common gamma chain gamma c  [ **IL2RG** ] , which is shared among receptors for multiple cytokines such as IL 2, IL 4, IL 7, IL 9 and IL 15  [ **IL15** ] .  Ref: 12069389 Am J Reprod Immunol, 2002
